# Successful Treatment of Acute on Chronic Mesenteric Ischaemia by Common Iliac to Inferior Mesenteric Artery Bypass

**DOI:** 10.1155/2015/962603

**Published:** 2015-09-01

**Authors:** D. N. Coakley, F. M. Shaikh, E. G. Kavanagh

**Affiliations:** Department of Vascular Surgery, University Hospital Limerick, Limerick, Ireland

## Abstract

Chronic mesenteric ischaemia is a rare and potentially fatal condition most commonly due to atherosclerotic stenosis or occlusion of two or more mesenteric arteries. Multivessel revascularisation of both primary mesenteric vessels, the celiac artery and superior mesenteric artery (SMA), is the current mainstay of treatment; however, in a certain cohort of patients, revascularisation one or both vessels may not be possible. Arteries may be technically unreconstructable or the patient may be surgically unfit for the prolonged aortic cross clamping times required. Here we present a case involving a 72-year-old woman with acute on chronic mesenteric ischaemia. She was a high risk surgical patient with severe unreconstructable stenotic disease of the SMA and celiac arteries. She was successfully treated with single vessel revascularisation of the inferior mesenteric artery (IMA) via a common iliac to IMA reversed vein bypass. At two-year follow-up, the graft remains patent and the patient continues to be symptom-free and is maintaining her weight.

## 1. Introduction

Chronic mesenteric ischaemia is an uncommon and challenging presentation in a vascular surgical unit. Diagnosis is difficult and is often delayed with patients frequently presenting at an advanced stage. It is generally caused by atherosclerotic stenosis of a least two mesenteric vessels and can be life threatening due to malnutrition or bowel infarction. The principle goal of treatment is to reduce postprandial pain, prevent bowel infarction, and allow the patient to resume a normal diet and regain lost weight. Due to the rarity of this disease, individual and institutional experience is lacking and presently there is no consensus regarding optimal treatment. Current opinion favors a multivessel surgical recanalisation of both major visceral arteries, the celiac artery and superior mesenteric artery (SMA) [1–4]. However, in a certain cohort of patients, this may not be technically feasible. Extensive atherosclerotic disease, complete occlusion, or small calibre arteries may render these vessels surgically unreconstructable. Patients are often malnourished with multiple comorbidities and thus are at high perioperative risk. They are poor surgical candidates for the prolonged aortic cross clamping required for extensive revascularisation. Also many patients have had previous abdominal surgeries or prior revascularisation attempts, resulting in scar tissue and adhesions which prevent safe access to these vessels. In such instances, the IMA may be the only vessel amenable to surgical intervention. We report a cachexic 72-year-old high risk patient with occluded SMA and highly stenotic small calibre celiac artery treated successfully with isolated revascularization of the IMA via the common iliac artery.

## 2. Case Report

**Figure 1:**
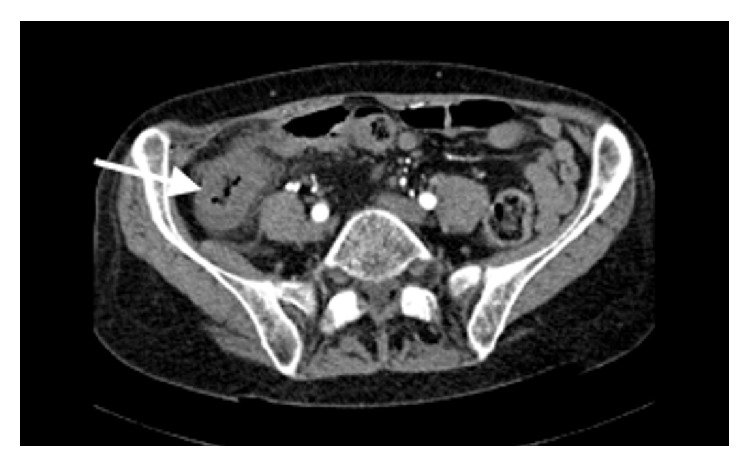
Abdominal CT scan with contrast demonstrating inflammatory changes of the pericolonic fat in the right colon particularly around the cecal area.

**Figure 2:**
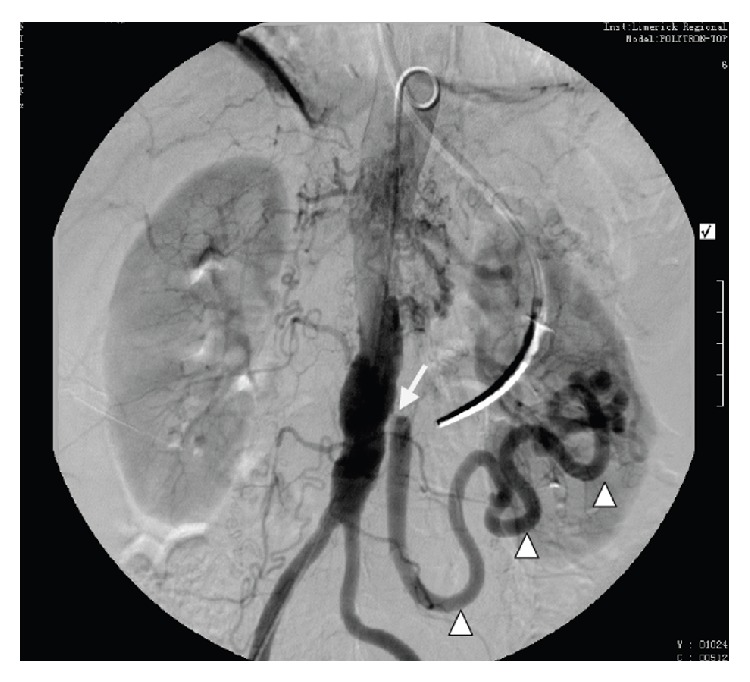
Preoperative aortogram with occlusion of the superior mesenteric artery and high grade stenosis of celiac and inferior mesenteric arteries (arrow). Note the markedly hypertrophied IMA which collateralises with the SMA via the marginal branch of Drummond (arrowheads) and the Arc of Riolan.

**Figure 3:**
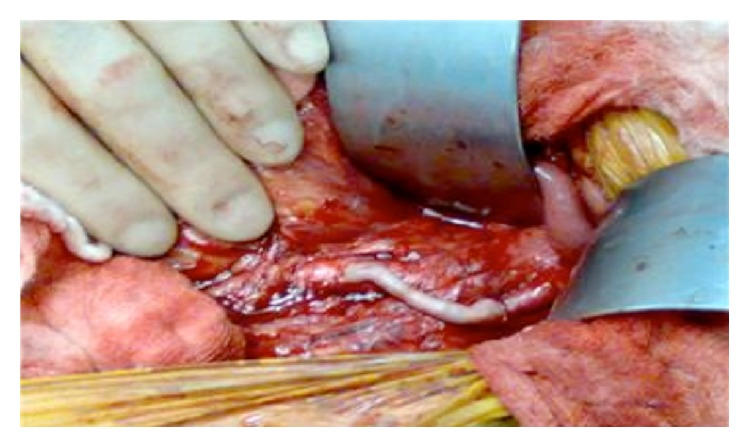
The right common iliac artery (RCI) and inferior mesenteric artery (IMA) were identified and isolated. The saphenous vein was harvested from the right leg and was reversed and anastomosed end to side from the proximal right common iliac artery to the proximal inferior mesenteric artery.

A 72-year-old female, exsmoker, presented to accident and emergency department with a 12-hour history of acute onset, sharp right sided abdominal pain associated with bilious vomiting. This occurred on a background of a recent aortic valve replacement (porcine valve) for critical aortic stenosis, currently warfarinised. Her past medical history included an open appendicectomy and open cholecystectomy. On examination, the patient was markedly cachexic, pulse was irregularly irregular at 140 beats per minute, and blood pressure and temperature were normal. Abdominal examination revealed localized peritonitis in the right iliac fossa. Blood results showed a markedly raised white cell count at 33.42 with an INR of 2.1. Hemoglobin, urea and electrolytes, amylase, and liver function tests and arterial blood gas were all normal. Abdominal CT scan with contrast demonstrated inflammatory changes of the pericolonic fat around the caecum (Figure 1). Marked narrowing of the celiac artery and complete occlusion of the SMA were noted. The inferior mesenteric artery was markedly hypertrophied with extensive collateral vessels to the bowel. No arterial thrombi or emboli were seen. The patient was consented for an urgent laparotomy. Intraoperatively a well-demarcated ischaemic caecum was noted with early signs of necrosis. Left colon, splenic flexure, and small intestine were normal. Right hemicolectomy was performed with primary anastomosis. This was combined with a defunctioning loop ileostomy, created in the right iliac fossa. Postoperatively the patient was commenced on a heparin infusion. Bowel function gradually returned, the stoma functioned normally, and normal diet was introduced. However, the postprandial abdominal pain, nausea, and vomiting persisted. Upon further questioning, she recounted an 18-month history of chronic prandial abdominal pain, vomiting, and weight loss. An aortogram (Figure 2) was performed which confirmed occluded SMA and small calibre celiac axis with high grade stenosis. A markedly hypertrophied inferior mesenteric artery was also found with over 90% stenosis at its origin. Extensive collaterals were seen via marginal branch of Drummond and the Arc of Riolan which supplied the small intestine. Owing to the two recent major operations and the fact that the patient was severely malnourished, she was deemed high risk for complete visceral revascularisation which would have involved prolonged aortic cross clamping. It was therefore decided for single vessel revascularisation of the IMA via a common iliac to IMA bypass. The patient was returned to theatre for an iliomesenteric bypass. A laparotomy was performed and the right common iliac artery and inferior mesenteric artery were identified and isolated. The long saphenous vein was harvested from the right leg and was reversed and anastomosed end to side from the proximal right common iliac artery to the proximal inferior mesenteric artery (Figure 3). Clamp time during this procedure was 20 minutes with the distal IMA continuously infused with saline/heparin. The patient was transferred to ICU and commenced on IV heparin. Postoperatively, patient made an uneventful recovery and was successfully introduced to a normal diet. After two weeks, she was discharged home on aspirin. At two-year follow-up the patient remains asymptomatic and had regained her lost weight. A duplex ultrasound revealed excellent flow in graft and a reversal of ileostomy is planned.

## 3. Discussion

Owing to the relative rarity of this condition, guidelines for treatment are lacking. There is no common consensus regarding the number of vessels to revascularise (one, two, or three) the technique of revascularisation (endarterectomy, antegrade, or retrograde bypass) or the type of bypass conduit (autologous or prosthetic). Current thinking favors multivessel revascularization of the principle arteries, namely, the SMA and celiac arteries to minimise the risk of recurrent ischaemia. Aortomesenteric bypass or transaortic mesenteric endarterectomy is the established methods of revascularization with five-year patency rate of over 85% [1]. Not all patients are suitable for revascularization of the SMA or celiac arteries as previously outlined. In this case, the patient had an occluded SMA with a highly stenotic small calibre celiac artery, rendering these vessels unsuitable for reconstruction. The patient was also severely malnourished at presentation with recent cardiac valve surgery; thus, the hemodynamic instability from prolonged aortic cross clamping was deemed too risky.

In this case the IMA was the only vessel amenable to revascularisation. The IMA was revascularised via a right common iliac to IMA bypass thus avoiding the need for prolonged aortic cross clamping. An autologous reversed saphenous venous graft was used as opposed to a synthetic graft, due to the increased risk of graft infection from the presence of the ileostomy. Transaortic endarterectomy of the IMA was not attempted here due to the high risk to the remaining visceral vessel. Endovascular repair of the IMA was also deemed too risky owing to the angle of takeoff, the high grade stenosis, and the risk of thrombosis of the remaining visceral vessel.

There is extensive collateral circulation between the three visceral arteries. SMA and IMA vasculature communicate via several anastomotic pathways, namely, the marginal artery of Drummond, lying proximal to the mesocolic border, and the Arc of Riolan which is formed centrally via branches of the middle and left colic arteries. The SMA anastomoses with the celiac vasculature via superior and inferior pancreaticoduodenal arteries and occasionally through the Arc of Bühler.

The above case illustrates that, in patients who are unfit for extensive revascularization or whose SMA or celiac arteries are unreconstructable, revascularization of the IMA is an acceptable alternative in alleviating symptoms, restoring small bowel function, and preventing bowel infarction.
